# Intensive versus standard blood pressure management after endovascular therapy in acute ischemic stroke: a systematic review and meta-analysis

**DOI:** 10.1097/MS9.0000000000004740

**Published:** 2026-01-20

**Authors:** Fatima Sajjad, Maria Qadri, Ayesha Arshad, Iqra Shahid, Asad Iqbal, Zoya Riaz, Umama Alam, Ammara Rehman, Gul Rukh Noreen, Muhammad Abdullah Ali, Zaryab Bacha, Fathimathul Henna, Malaika Usman, Umama Rehman, Kamil Ahmad Kamil

**Affiliations:** a Department of Medicine, Khyber Medical College, Peshawar, Pakistan; b Department of Medicine, Jinnah Sindh Medical University, Karachi, Pakistan; c Department of Public Health, Dow Medical College, Karachi, Pakistan; d Department of Medicine, King Edward Medical University, Lahore, Pakistan; e Department of Medicine, Bacha Khan Medical College, Mardan, Pakistan; f Department of Medicine, Rawalpindi Medical University, Rawalpindi, Pakistan; g Department of Medicine, Khyber Teaching Hospital, Peshawar, Pakistan; h Department of Medicine, Dubai Medical College for Girls, Dubai, United Arab Emirates; i Department of Medicine, Mirwais Regional Hospital, Kandahar, Afghanistan

**Keywords:** acute ischemic stroke, blood pressure management, endovascular thrombectomy, intensive blood pressure control, standard care

## Abstract

Endovascular thrombectomy (EVT) has improved the therapy of acute ischemic stroke, patients still have functional impairments after reperfusion. This updated meta-analysis assesses whether intense blood pressure (BP) management following successful EVT enhances results and reduces problems associated with reperfusion. PubMed, Embase, and Cochrane databases were systematically searched using relevant keywords from inception until July 2025. A total of five studies were included after final screening. Outcomes were reported as excellent clinical outcomes, functional independence, all-cause mortality at 90 days, any intracranial hemorrhage within 24 h, etc. Interstudy heterogeneity was assessed using *I*^2^ and *X*^2^ statistics (*I*^2^ > 50% = significant heterogeneity). Statistical calculations used Review Manager 5.4.1, with a *P*-value of <0.05 indicating statistical significance. A moderate but substantial improvement in neurological outcomes (mean National Institutes of Health Stroke Scale difference = 1.07; *P* = 0.03) and functional independence (mRS modified Rankin Scale 0–2) was both markedly enhanced by intensive BP control (RR = 0.82; 95% CI: 0.74–0.91; *P* = 0.0002). It did not, however, have a significant effect on all-cause death at 90 days, symptomatic or any cerebral bleeding, stroke recurrence, or excellent clinical outcomes (mRS 0–1). A significant correlation was seen between a greater incidence of hypotensive episodes and severe BP lowering (RR = 1.77; *P* < 0.00001). It also raises the risk of hypotension without reducing the danger of hemorrhage or death. Following EVT, intense BP control may enhance early neurological results and functional independence, but it also raises the risk of hypotension without reducing death or hemorrhage.

## Introduction

Acute ischemic stroke is a major worldwide health concern, and endovascular thrombectomy (EVT) has become a crucial therapy advancement that has significantly changed the stroke services’ administration and coordination^[1,2]^. Blood flow to an ischemic region of the brain, known as the penumbral tissue, can be successfully restored (i.e., reperfusion) by removing the site of obstruction or clot in an artery by endovascular insertion of a stent retriever, aspiration, or a combination of such devices (i.e., recanalization). Adjuvant techniques are being used to protect or sustain penumbral tissue from reperfusion injury following EVT because many patients have poor functional recovery even after achieving a good radiological result and because there is still a high risk of symptomatic intracranial hemorrhage (sICH) and other types of reperfusion injury^[3–5]^.

**HIGHLIGHTS**

- Intensive versus standard BP control post-thrombectomy was systematically reviewed.
- Meta-analysis shows impact on outcomes in acute ischemic stroke patients.
- No external funding; transparent methods with PRISMA 2020 compliance.
- Provides guidance for BP targets after endovascular stroke therapy.

Due to its frequent elevation, ease of modification, and observational studies that have unequivocally demonstrated its prognostic importance in acute ischemic stroke with endovascular therapy, blood pressure (BP) may be a modifiable factor to avoid reperfusion damage. BP, the extent of the ischemic penumbra, the effectiveness of collateral cerebral blood flow, the degree of reperfusion, and the clinical consequences of acute ischemic stroke are likely to have complex interactions^[6]^. However, there is now more support for the use of EVT, a desire to lower the risk of ischemia-reperfusion injury, and compelling evidence linking BP at presentation to later clinical outcomes. These factors have caused a shift in research and practice toward more stringent systolic BP (SBP) control^[7–9]^. Despite the fact that over 80% of patients who had received EVT were able to regain their functional dependence, over half of these patients continued to be functionally dependent following therapy. According to observational studies, there was a correlation between the functional result and BP following EVT. While too low BP might worsen ischemic injury, elevated BP during recanalization may cause perfusion damage. Despite the current stroke guidelines’ recommendation to keep BP below 180/105 mmHg for 24 h following recanalization, there is not enough data to determine the ideal BP goals^[10]^. The guidelines continue to advise maintaining lower BP values after EVT, comparable with those for patients, in the lack of randomized data. Via intravenous thrombolysis during an acute ischemic stroke^[11,12]^, the aim of this updated meta is to assess the safety and efficacy of intensive BP management compared to less intensive treatment in patients with successful reperfusion following EVT. This manuscript adheres to TITAN guidelines^[13]^.

## Methods

### Study design and protocol registration

This systematic review adhered to the guidelines set by the Cochrane Collaboration^[14]^ and the Preferred Reporting Items for Systematic Reviews and Meta-Analysis (PRISMA) framework^[15]^. It encompassed the study design, stepwise implementation, analysis, and presentation of findings. Additionally, the study protocol was registered in the International Prospective Register of Systematic Reviews (PROSPERO). The manuscript follows AMSTAR guidelines.

### Search strategy and databases

An electronic search of PubMed, Embase, and Cochrane was conducted, covering all available entries from their inception to July 2025, without any language restrictions. The following keywords were used: “Acute Ischemic Stroke,” “Intensive Blood Pressure Management,” “Endovascular Thrombectomy,” and “Standard Blood Pressure Management.”

### Study selection and eligibility criteria

All studies identified through the online search were imported into the Rayyan software for screening, and duplicate records were removed. The remaining studies were initially screened based on their titles and abstracts. Full-text articles were retrieved for further assessment if either reviewer found the abstract potentially relevant. Two independent reviewers (U.A. and F.S.) evaluated the eligibility of each study according to predefined inclusion criteria. Any disagreements were resolved through discussion and consultation with a third reviewer (Z.B.).

Studies were included if they met the following criteria: (1) acute ischemic stroke; (2) intensive SBP targets ranging from <120 to <160 mmHg as intervention; (3) standard or conventional targets ranging from 130 to ≤185 mmHg as control; (4) randomized controlled trials (RCTs); and (5) reported at least one relevant outcome. The included RCTs differed in their definitions of “intensive” and “standard” SBP targets. This variability most likely reflects variations in patient populations and institutional protocols. Data were reviewed carefully to ensure consistency and accuracy across studies.

Exclusion criteria included: (1) retrospective and prospective observational studies, including cohort studies, case control, case series, and cross-sectional studies; (2) overlapping populations, defined by shared institutions and recruitment periods; (3) populations outside the scope of interest; (4) republished literature; (5) protocols without reported results; (6) reviews, abstracts, background articles, expert opinions, or *in vivo*/*in vitro* studies; (7) duplicate data from the same clinical trial; or (8) absence of a comparator group.

### Data extraction and outcomes

Two authors (Z.R. and U.A.) extracted data from the included studies into an Excel sheet using a pre-piloted form. Baseline data included age, male, hypertension, diabetes, hyperlipidemia, atrial fibrillation, coronary artery disease, current smoking, previous smoking, SBP and diastolic BP (DBP) prior to endovascular thrombectomy, baseline National Institutes of Health Stroke Scale (NIHSS), modified thrombolysis in cerebral infarction, and intravenous thrombolysis. Outcomes were categorized into primary and secondary outcomes. The primary outcomes of this study included excellent clinical outcome (mRS 0–1) and functional independence (mRS 0–2). The secondary outcomes comprised all-cause mortality at 90 days, any intracerebral hemorrhage within 24 h, hypotensive event within 24 h, NIHSS, sICH within 24 h, and stroke recurrence. See Supplemental Digital Content Table S1, available at: http://links.lww.com/MS9/B81, for a detailed definition of outcomes.

### Quality assessment

The quality of the included studies was assessed using appropriate tools based on their study design. For RCTs, the Revised Cochrane Risk of Bias Tool for Randomized Trials (RoB 2)^[16]^ was used, evaluating bias across five domains: randomization process, deviations from intended interventions, missing outcome data, measurement of outcomes, and selection of reported results. Each study’s overall risk of bias was categorized as low, some concerns, or high risk.

### Certainty of evidence

The Grading of Recommendations, Assessment, Development, and Evaluation (GRADE) tool was employed by two independent authors (A.I.K. and M.A.A.) using the GRADEpro Guideline Development Tool^[17]^ to evaluate the level of certainty of the evidence in this meta-analysis, with categorizations ranging from high to very low^[18]^.

### Statistical analysis and sensitivity analysis

Review Manager 5.4 was used to perform statistical analysis. Treatment effects for binary outcomes were compared using a pooled risk ratio (RR) with 95% confidence intervals (CI), while continuous outcomes were analyzed using mean differences (MDs) with 95% CI. The Cochran *Q* test and *I*^2^ statistics were used to assess heterogeneity, with *P*-values < 0.10 and *I*^2^ > 50% considered indicative of significant heterogeneity^[19]^. The DerSimonian and Laird random-effects model was applied to all outcomes^[20]^. A *P*-value of <0.05 indicates statistical significance for clinical endpoints. The stability of the pooled estimates was assessed through a leave-one-out analysis, where each study was sequentially removed, and the remaining dataset was re-analyzed to ensure that no single study unduly influenced the aggregated effect sizes.

### Trial sequential analysis

Trial sequential analysis (TSA) was performed for primary outcomes to assess the robustness of our results regarding type 1 and type 2 errors^[21]^. Required information size (RIS) was calculated considering a type 1 error of 5% and a power of 80%. *Z*-score was calculated to examine the benefit, harm, or futility of intensive BP management compared to standard BP management in patients with acute ischemic stroke after EVT^[22]^.

## Results

A total of five RCTs were included in this meta-analysis, evaluating the impact of intensive versus standard SBP targets in patients with acute ischemic stroke after EVT.

### Study selection process

**Figure 1.**
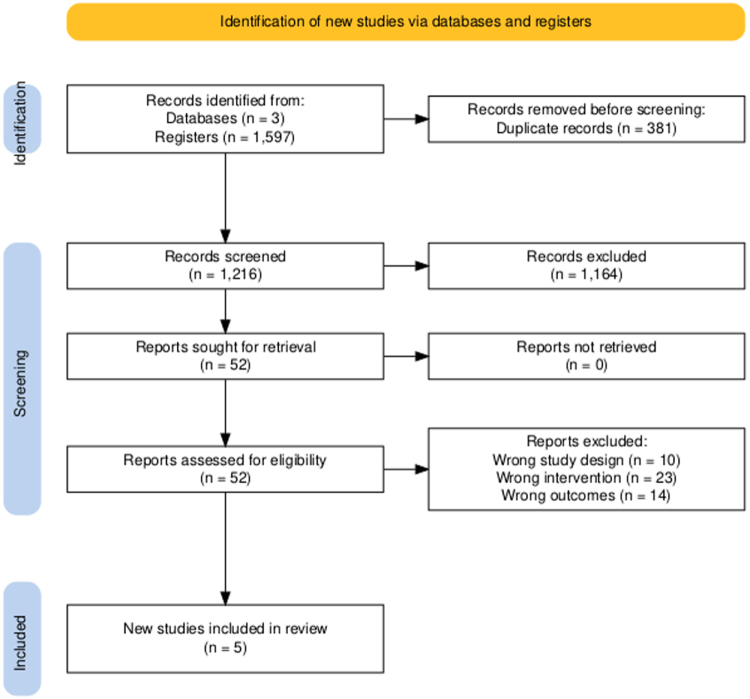
PRISMA flowchart.

A total of 1600 records were identified through database and register searches. After removing 381 duplicates, 1216 records were screened. Out of these, 52 full-text records were assessed for eligibility, and 47 were excluded due to inappropriate study design, interventions, or outcomes. Ultimately, five studies met the inclusion criteria^[6,10,23–25]^ and were included in the final analysis (Fig. 1).

### Risk of bias assessment

The overall risk of bias, assessed using the ROB 2 tool, showed that the majority of domains across included trials were rated as low risk. However, “bias due to deviations from intended interventions” and the “overall risk of bias” raised some concerns in some of the studies. Most other domains demonstrated consistently low risk, such as the randomization process, outcome data, and outcome measurement, as mentioned in Supplemental Digital Content Figure S1, available at: http://links.lww.com/MS9/B81 and Supplemental Digital Content Figure S2, available at: http://links.lww.com/MS9/B81.

### Study characteristics

The mean age across the studies ranged from 67 to 76 years, with similar distributions between intervention groups. The proportion of male participants ranged from 30 to 63%, with no consistent difference between groups. Hypertension was highly prevalent, reported in 64–80% of the participants. Diabetes mellitus ranged from 20 to 42.2%, while hyperlipidemia varied substantially. Atrial fibrillation was commonly reported, where available, affecting up to 61.5% of patients. Coronary artery disease was inconsistently reported, with rates between 10.9 and 22.5%. Smoking prevalence ranged from 14 to 27.5%, though data were missing in some studies. Previous stroke was reported in 13–34% of patients. SBP prior to EVT, when reported, ranged from 150.8 to 158.1 mmHg, with DBP ranging from 85 to 89.5 mmHg. Use of intravenous thrombolysis ranged from 28.4 to 54%, with similar proportions across groups within each study. Studies were conducted in France, the US, South Korea, and China. Detailed baseline characteristics are mentioned in Tables 1 and 2.

**Table 1:** Study characteristics

Author, year	Recruitment period	Country	Type of study	Intervention	Control	Sample size		Mean follow-up	
						Intensive SBP target	Standard SBP target	Intensive SBP target	Standard SBP target
Mazighi 2021	Jun 2017–Sept 2019	France	RCT	Intensive systolic blood pressure (SBP) target: 100–129 mmHg	Standard systolic blood pressure (SBP) target: 130–185 mmHg	158	160	115 days	111 days
Mistry 2023	Jan 2020–Feb 2022	United States	RCT	Intensive SBP control with three target groups: 40 to <140 mmHg, <160 mmHg	Standard SBP target ≤180 mmHg	40	80	90 days	90 days
Nam 2023	Jun 2020–Nov 2022	South Korea	RCT	Intensive BP management (SBP target <140 mmHg)	Conventional BP management (SBP target 140–180 mmHg)	155	151	3 months	3 months
Yang 2022	Jul 2020–Mar 2022	China	RCT	Intensive BP management (SBP target <120 mm H g)	Less intensive BP management (SBP target 140–180 mm H g)	404	406	90 days	90 days
Zhang 2025	Oct 2022–Mar 2024	China	RCT	Intensive BP management (target SBP <130 mmHg for 24 h post-EVT)	Standard BP management (target SBP <180 mmHg for 24 h post-EVT)	183	200	90 days	90 days

EVT, endovascular thrombectomy; RCT, randomized control trials; SBP, systolic blood pressure.

**Table 2:** Patient characteristics

Author, year	Mazighi 2021		Mistry 2023		Nam 2023		Yang 2022		Zhang 2025	
	Intensive SBP	Standard SBP	Intensive SBP	Standard SBP	Intensive SBP	Standard SBP	Intensive SBP	Standard SBP	Intensive SBP	Standard SBP
Age (years) Mean (SD)	76 (14.2)	73.(14.9)	72 (12.3)	67 (13.8)	73.2 (12.1)	72.9 (10.8)	68 (12)	67 (12)	72 (11)	72 (10)
Male (%)	51	45	39	50	59	60	61	63	49	53
Hypertension (%)	70	71	75	80	78	75	66	64	74	67
Diabetes (%)	22	21	34	33	42	42	20	20	27	23
Hyperlipidemia (%)	39	35	76	85	39	37	3	3	11	15
Atrial fibrillation (%)	NR	NR	40	53	50	47	21	24	62	61
Coronary artery disease (%)	NR	NR	NR	NR	12	11	13	14	23	22
Current smoking (%)	14	16	25	25	25	20	NR	NR	24	25
Previous stroke (%)	16	13	NR	NR	23	20	26	34	NR	NR
SBP prior to EVT (mmHg) Mean (SD)	155 (26)	152 (25)	151 (23.8)	146 (23)	NR	NR	158.1 (25)	158.7 (23)	150.8 (25.51)	151.9 (23.30)
DBP prior to EVT (mmHg) Mean (SD)	86 (18)	85 (15)	85.8 (16.8)	86.6 (19.6)	NR	NR	89.4 (16)	89.5 (15)	85.92 (17.57)	86.20 (15.25)
Baseline NIHSSMean (SD)	16.5 (5.9)	16.6 (5.2)	17.7 (7.6)	14 (4.6)	13 (6)	12 (7)	15 (7.4)	15 (7.4)	15.6 (5.2)	15 (4.4)
mTICI 2b (%)	44	48	41	43	NR	NR	NR	NR	19	18
mTICI 3 (%)	56	52	45	40	NR	NR	NR	NR	79	82
Intravenous thrombolysis (%)	54	52	45	45	28	37	32	28	42	43
ICA vessel occlusion (%)	NR	NR	20	13	NR	NR	NR	NR	39	33
M1 vessel occlusion (%)	NR	NR	70	58	NR	NR	NR	NR	44	56
M2 vessel occlusion (%)	NR	NR	18	38	NR	NR	NR	NR	15	11

DBP, diastolic blood pressure; ICA, internal carotid artery; M1, first segment of middle cerebral artery; M2, second segment of middle cerebral artery; mTICI, modified thrombolysis in cerebral infarction; NIHSS, National Institutes of Health Stroke Scale; SBP, systolic blood pressure.

### Publication bias

#### MRS 0–1

Egger’s regression test (intercept = 1.733, 95% CI: −0.23 to 3.69, *P* = 0.181) indicates an absence of significant publication bias; the funnel plot exhibits no substantial asymmetry. These findings enhance the credibility of this safety outcome and suggest a minimal risk of selective reporting (Supplemental Digital Content Figure S3, available at: http://links.lww.com/MS9/B81).

#### MRS 0–2

Egger’s regression test (intercept = 1.415, 95% CI: −0.74 to 3.57, *P* = 0.2888) indicates an absence of significant publication bias; the funnel plot exhibits no substantial asymmetry. These findings enhance the credibility of this safety outcome and suggest a minimal risk of selective reporting (Supplemental Digital Content Figure S4, available at: http://links.lww.com/MS9/B81).

#### All-cause mortality

Egger’s regression test (intercept = −0.317, 95% CI: −2.76 to 2.13, *P* = 0.816) indicates an absence of significant publication bias; the funnel plot exhibits no substantial asymmetry. These findings enhance the credibility of this safety outcome and suggest a minimal risk of selective reporting (Supplemental Digital Content Figure S5, available at: http://links.lww.com/MS9/B81).

#### Symptomatic intracranial hemorrhage

Egger’s regression test (intercept = 0.652, 95% CI: −1.56 to 2.86, *P* = 0.603) indicates an absence of significant publication bias; the funnel plot exhibits no substantial asymmetry. These findings enhance the credibility of this safety outcome and suggest a minimal risk of selective reporting (Supplemental Digital Content Figure S6, available at: http://links.lww.com/MS9/B81).

#### National Institutes of Health Stroke Scale

The funnel plot exhibits notable asymmetry, suggesting possible small-study effects or publication bias, with absent data primarily on the left side, implying an absence of studies revealing smaller or less advantageous effect sizes. Egger’s regression test (intercept = 1.93, 95% CI: 1.18–2.68, *P* = 0.0375) indicates a statistically significant publication bias. This data suggests a danger of selective reporting, indicating that trials with less favorable functional outcomes may be under-represented. Consequently, caution is necessary when analyzing this result (Supplemental Digital Content Figure S7, available at: http://links.lww.com/MS9/B81).

#### Hypotensive events

Egger’s regression test (intercept = 0.606, 95% CI: −0.35 to 1.56, *P* = 0.430) indicates an absence of significant publication bias; the funnel plot exhibits no substantial asymmetry. These findings enhance the credibility of this safety outcome and suggest a minimal risk of selective reporting (Supplemental Digital Content Figure S8, available at: http://links.lww.com/MS9/B81).

#### Intracranial hemorrhage

Egger’s regression test (intercept = 0.194, 95% CI: −0.97 to 1.36, *P* = 0.765) indicates an absence of significant publication bias; the funnel plot exhibits no substantial asymmetry. These findings enhance the credibility of this safety outcome and suggest a minimal risk of selective reporting (Supplemental Digital Content Figure S9, available at: http://links.lww.com/MS9/B81).

#### Stroke recurrence

Egger’s regression test (intercept = 1.45, 95% CI: −2.31 to 5.20, *P* = 0.334) indicates an absence of significant publication bias; the funnel plot exhibits no substantial asymmetry. These findings enhance the credibility of this safety outcome and suggest a minimal risk of selective reporting (Supplemental Digital Content Figure S10, available at: http://links.lww.com/MS9/B81).

### Outcomes

#### Primary outcomes

##### Excellent clinical outcomes mRS (0–1)

**Figure 2.**
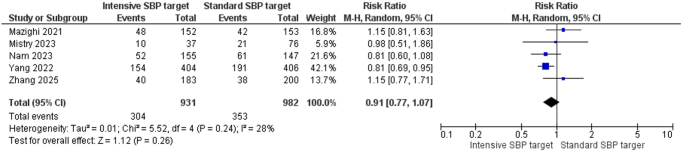
Forest plot of excellent clinical outcome (mRS 0–1).

The pooled analysis showed no statistically significant difference in the rates of excellent clinical outcomes (mRS 0–1), between the intensive and standard care SBP target groups. The combined RR was 0.91 [95% CI, 0.77–1.07], with low statistical heterogeneity across the studies (*I*^2^ = 28%, *P* = 0.24). The test for overall effect was not significant (*Z* = 1.12, *P* = 0.26), suggesting that intensive BP lowering did not significantly improve the likelihood of achieving excellent clinical status at follow-up (Fig. 2).

##### Functional independence mRS (0–2)

**Figure 3.**
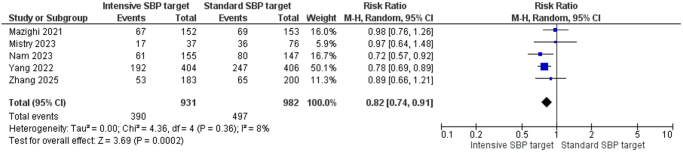
Forest plot of functional independence (mRS 0–2).

In contacts, the intensive SBP target group demonstrated a significantly higher rate of functional independence mRS (0–2) compared to the standard SBP group. The pooled RR was 0.82 [95% CI, 0.74–0.91], favoring the intensive target group. Heterogeneity was minimal (*I*^2^ = 8%, *P* = 0.36), and the result was statistically significant (*Z* = 3.69, *P* = 0.0002), indicating a strong association between intensive BP lowering and improved functional independence (Fig. 3).

#### Secondary outcomes

##### All-cause mortality at 90 days

**Figure 4.**
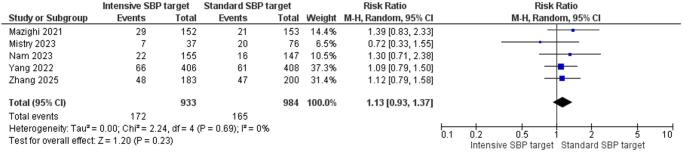
Forest plot of all-cause mortality at 90 days.

Five studies reported data on all-cause mortality at 90 days, consisting of a total of 1917 participants (933 in the intensive SBP target group and 984 in the standard SBP target group). The pooled analysis demonstrated no significant difference in mortality amongst the two groups (RR = 1.13, 95% CI: 0.93–1.37, *Z* = 1.20, *P* = 0.23). There was no observed heterogeneity amongst the studies (*I*^2^ = 0%, *P* = 0.69), suggesting consistent findings across trials (Fig. 4).

##### Any intracranial hemorrhage within 24 h

**Figure 5.**
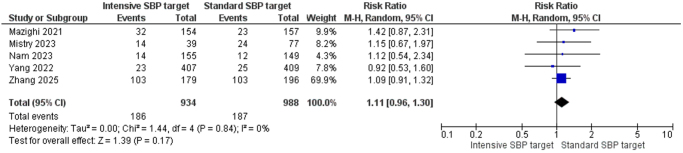
Forest plot of any intracranial hemorrhage within 24 h.

Five studies involving 1922 participants (934 in the intensive SBP group and 988 in the standard group) reported intracranial hemorrhage (ICH) within 24 h. No statistically significant difference was observed between the groups (RR = 1.11, 95% CI: 0.96–1.30, *Z* = 1.39, *P* = 0.17), and no heterogeneity was present (*I*^2^ = 0%, *P* = 0.84) (Fig. 5).

##### Hypotensive events within 24 h

**Figure 6.**
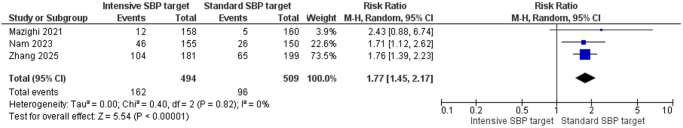
Forest plot of hypotensive events within 24 h.

Three studies (*n* = 1003; 494 intensive SBP group vs. 509 standard SBP group) assessed hypotensive events. Intensive SBP target groups were associated with a significantly increased risk of hypotension (RR = 1.77, 95% CI: 1.45–2.17, *Z* = 5.54, *P* < 0.00001). Heterogeneity was absent (*I*^2^ = 0%, *P* = 0.82) (Fig. 6).

##### Change in neurological deficit (NIHSS)

**Figure 7.**
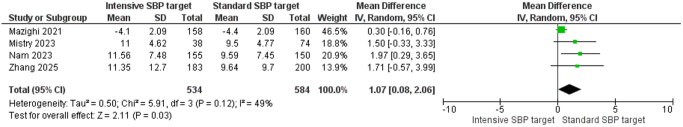
Forest plot of change in neurological deficit (NIHSS).

Four studies reported the MD in NIHSS (National Institutes of Health Stroke Scale) between the two SBP target groups. The pooled analysis showed a small, but statistically significant improvement in NIHSS score in the intensive SBP target group, with a MD of 1.07 [95% CI: 0.08–2.06], along with moderate heterogeneity (*I*^2^ = 49%, *P* = 0.12). The overall effect was statistically significant (*Z* = 2.11, *P* = 0.03), indicating that intensive SBP lowering may be associated with better early neurological outcomes (Fig. 7).

##### Symptomatic intracranial hemorrhage within 24 h

**Figure 8.**
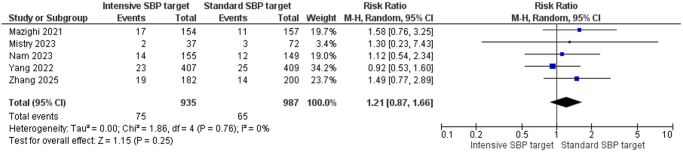
Forest plot of symptomatic intracerebral hemorrhage within 24 h.

Five studies (*n* = 1922; 935 intensive SBP group vs. 987 standard SBP group) assessed the incidence of sICH within 24 h of treatment. The pooled RR was 1.21 [95% CI: 0.87–1.66], suggesting no statistically significant difference between the intervention and control groups. There was no heterogeneity (*I*^2^ = 0%, *P* = 0.76), and the test for overall effect was not significant (*Z* = 1.15, *P* = 0.25), showing a similar risk of early sICH in both groups (Fig. 8).

##### Stroke recurrence

**Figure 9.**
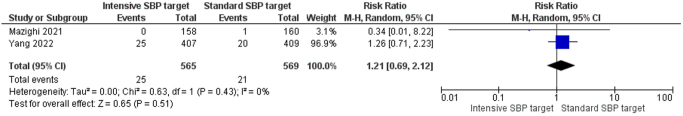
Forest plot of stroke recurrence.

Data on stroke recurrence were reported by two studies (*n* = 1922; 565 intensive SBP group vs. 569 standard SBP group). The pooled analysis showed no significant difference between the groups, with an RR of 1.21 [95% CI: 0.69–2.12]. Heterogeneity was absent (*I*^2^ = 0%, *P* = 0.43), and the result was not statistically significant (*Z* = 0.65, *P* = 0.51). These findings suggest that intensive SBP reduction did not significantly affect the risk of stroke recurrence in the patients (Fig. 9).

### Trial sequential analysis

#### Excellent functional outcome (mRS 0–1)

The *Z*-curve initially dips below the null line (*Z* = 0) but later rises above the upper significance boundary, indicating a statistically significant benefit. TSA suggests that intensive BP management may significantly improve excellent functional outcomes (mRS 0–1), as the cumulative evidence has crossed the efficacy boundary (Supplemental Digital Content Figure S11, available at: http://links.lww.com/MS9/B81).

#### Functional independence (mRS 0–2)

The *Z*-curve in this figure does not cross either the efficacy. The present sample size is 1115 patients, compared with an RIS of 1221 patients. TSA findings indicate that the current evidence is close to reaching the required threshold but remains inconclusive. Additional research may clarify the true effect of intensive BP management on functional independence (Supplemental Digital Content Figure S12, available at: http://links.lww.com/MS9/B81).

#### All-cause mortality

In this analysis, the *Z*-curve remains within the futility boundaries and does not cross the significance thresholds. The current cumulative sample size is 1917 patients, while the RIS is estimated at 16 323 patients. This indicates that the available evidence is currently inconclusive and additional data are needed to determine whether intensive SBP targets have an impact on all-cause mortality (Supplemental Digital Content Figure S13, available at: http://links.lww.com/MS9/B81).

#### Any intracranial hemorrhage

The *Z*-curve trends downward, staying below the null line and approaching the lower significance boundary. With a current sample size of 1057 patients, this result suggests a possible increase in ICH associated with intensive SBP targets. However, the lack of statistical confirmation means the observed trend should be interpreted with caution. (Supplemental Digital Content Figure S14, available at: http://links.lww.com/MS9/B81).

#### Hypotensive events

The *Z*-curve shows a steep decline below the lower significance boundary. This finding confirms a statistically significant increase in hypotensive events among patients managed with intensive SBP targets. TSA therefore supports the conclusion that standard BP management is safer with regard to hypotensive complications (Supplemental Digital Content Figure S15, available at: http://links.lww.com/MS9/B81).

#### NIHSS (neurological deficit)

The *Z*-curve for this outcome remains below the significance boundaries. The present sample size is 118 patients, compared with an RIS of 1971 patients. TSA findings suggest that there is insufficient evidence to confirm a significant effect of intensive systolic or SBP targets on NIHSS outcomes, highlighting the need for further clinical trials (Supplemental Digital Content Figure S16, available at: http://links.lww.com/MS9/B81).

#### Symptomatic intracranial hemorrhage

In this figure, the *Z*-curve lies well below the null line, favoring the standard SBP target. The current sample size includes 1922 patients, while the RIS is 10 246 patients. The analysis indicates a strong trend toward an increased risk of sICH with intensive SBP targets. However, the RIS has not yet been achieved, and conclusions remain preliminary (Supplemental Digital Content Figure S17, available at: http://links.lww.com/MS9/B81).

### Certainty of evidence

The GRADE approach, using the GRADEpro Guideline Development Tool, was employed to assess the certainty of evidence. A detailed assessment is shown in Supplemental Digital Content Table S2, available at: http://links.lww.com/MS9/B81.

## Discussion

This updated meta-analysis synthesizes the results of five RCTs assessing the safety and efficacy of intensive versus standard SBP targets in patients with acute ischemic stroke undergoing EVT. Compared to the previous meta-analysis by Jie *et al* (2025)^[26]^, which included only 4 RCTs and 1556 participants, this updated meta-analysis incorporates a fifth trial, thereby expanding the patient pool to 1937. Our findings demonstrate a complex risk-benefit profile that varies across different outcome measures. The analysis revealed that intensive SBP management significantly improved the rate of functional independence (mRS 0–2) and resulted in a small but statistically significant improvement in neurological deficit measured by NIHSS score. However, there were no significant differences between the two treatment approaches in terms of excellent clinical outcomes (mRS 0–1), all-cause mortality, cerebral hemorrhage episodes, or stroke recurrence. The significantly higher probability of hypotensive episodes with intensive SBP control (RR = 1.77, *P* < 0.00001) was a clinically relevant finding that highlighted an important safety concern. According to TSA, there is strong evidence for functional independence and hypotensive episodes, but further research is needed to draw firm judgments about other outcomes. Moreover, while the previous meta-analysis focused primarily on three endpoints: excellent functional outcomes (mRS 0–1), functional independence (mRS 0–2), and mortality, we expanded our scope to include five secondary outcomes: any ICH, sICH within 24 h, hypotensive events within 24 h, NIHSS improvement, and stroke recurrence.

Furthermore, the conflicting results between our functional outcome measures, a notable improvement in mRS 0–2 but not in mRS 0–1, reflect a persistent debate in the literature about the best BP levels in acute cerebrovascular situations. Our findings are consistent with the ENCHANTED experiment^[27]^, which showed that compared to normal guidelines (SBP target <180 mmHg), intense BP lowering (SBP target 130–140 mmHg) had comparable functional outcomes. In contrast, a *post hoc* analysis of the INTERACT2 study^[28]^ revealed that, in comparison to standard treatment (SBP <180 mmHg), intensive BP control (SBP <140 mmHg) was linked to better functional outcomes. The difference between mRS 0–2 and mRS 0–1 results may indicate that while intense BP control is beneficial enough to help patients transition from severe disability to functional independence, it may not always encourage full neurological recovery. This pattern was also seen in the ATACH-2 study^[29]^, where there was a trend toward better mRS 0–2 outcomes but no increase in the percentage of patients with mRS 0–1 at 90 days.

Moving on to the primary outcomes, Jie *et al* (2025)^[26]^ reported no significant statistical difference in the functional outcomes between the intervention and control groups. However, our analyses found that intensive BP lowering significantly improved functional independence (mRS 0–2), with minimal heterogeneity. This contradicts their findings, likely due to updated data and broader outcome modeling. The improvement in functional independence (mRS 0–2), despite the lack of effect on mRS (0–1), suggests that intensive BP control may not necessarily lead to full recovery, but may help prevent moderate disability. Previous meta-analysis and observational studies reported that elevated BP increased the risk of ICH and worse outcomes in patients with successful reperfusion after EVT^[30–32]^. This is because reperfused vessels are more vulnerable to high BP due to ischemic and mechanical damage from EVT, which disrupts the blood-brain barrier and increases the risk of edema and hemorrhagic transformation. This supports the rationale for intensive BP control to minimize reperfusion injury and improve functional outcomes^[33–36]^. Hence, it is important to recognize that hemorrhagic complications and mortality following EVT are influenced by multiple factors beyond BP management. Procedural factors, including vessel manipulation and reperfusion injury, as well as patient-specific factors such as comorbidities, infarct size, and collateral circulation, can significantly affect outcomes. Therefore, while intensive SBP lowering may reduce reperfusion-related injury, the risk of hemorrhage and mortality cannot be entirely attributed to BP management alone. This highlights the need to interpret our findings in the context of these confounding factors and underscores the multifactorial nature of outcomes after thrombectomy.

Additionally, we included changes in NIHSS scores to measure early improvement in neurological function, which were not assessed by Jie *et al* (2025)^[26]^. Improved NIHSS results show the positive effect of intensive BP lowering on early neurological stabilization and suggest potential early neuroprotective benefits of the intervention. A study by You *et al* (2019) showed that participants with a higher SBP were at a higher risk of early or late neurological deterioration^[37]^. However, patients also usually use anti-hypertensive medications after EVT, which may have neuroprotective benefits^[38]^. The results of the INTERACT2 study^[39]^, which showed improved functional recovery with early intensive BP lowering in acute intracerebral hemorrhage, are consistent with the significant improvement in NIHSS scores with intensive SBP management (MD 1.07, *P* = 0.03). This is in contrast to the ENOS experiment^[40]^, which demonstrated that glyceryl trinitrate therapy for BP control did not significantly alter the modified Rankin Scale at day 90. According to a meta-analysis by Tsivgoulis *et al* (2014)^[41]^, intensive BP lowering (SBP <140 mmHg) was linked to decreased hematoma growth, which may be the mechanism underlying better neurological outcomes in intracerebral hemorrhage. In a similar vein, Qureshi *et al* (2009)^[42]^ suggested that early, strict BP management could prevent the development of perihematomal edema, enhancing neurological results. Our meta-analysis identifies an increased risk of hypotensive events, a finding not emphasized in earlier work. Although these episodes did not lead to worse outcomes in our data, they remain clinically relevant because reduced BP can compromise cerebral perfusion, especially in severe stroke or impaired autoregulation^[43–45]^. The higher risk observed in our analysis (RR = 1.77, *P* < 0.00001) aligns with the ATACH-2 trial, which also reported more hypotensive episodes in the intensive-treatment group (2.0 vs. 0.2%)^[29]^. However, their clinical impact remains uncertain, as neither our analysis nor Maier *et al* (2017) found an association with increased mortality or significant functional decline^[46]^. In contrast to some earlier studies, we found no significant difference in sICH between groups (RR = 1.21, *P* = 0.25). While Berge *et al*^[47]^ observed no significant difference in sICH rates between BP techniques in acute ischemic stroke.

The neutral effect on all-cause mortality observed in our analysis (RR = 1.13, *P* = 0.23) aligns with findings from prior high-quality randomized trials. In INTERACT2, intensive versus standard SBP reduction did not yield a statistically significant mortality difference (12.0 vs. 13.6%, *P* = 0.37)^[28]^, a result further supported by the ATACH-2 trial, which likewise demonstrated comparable mortality between treatment groups (6.6 vs. 6.8%, *P* = 0.94)^[29]^. Nonetheless, the possibility of mortality benefit in select patient subgroups has been suggested by Woodward *et al*^[48]^ reported that individuals presenting with higher baseline SBP may derive greater benefit from intensive BP lowering. With respect to stroke recurrence, our findings are consistent with the previous study^[49]^, which concluded that evidence remains insufficient to support a protective effect of acute intensive BP control on early recurrent stroke. In contrast, long-term secondary prevention data indicate a different trend. The SPS3 trial demonstrated that targeting a SBP <130 mmHg significantly reduced recurrent stroke risk among patients with lacunar infarcts^[50]^, underscoring that the benefits of intensive BP lowering may depend on timing, stroke subtype, and chronic rather than hyperacute management contexts.

Hence, although our meta-analysis did not find statistically significant differences in some outcomes, such as mortality, sICH, and stroke recurrence, between the intensive and standard SBP target groups, previous studies have reported on these outcomes. Many previous studies report a reduction in the risk of ICH with intensive BP control^[51–56]^. In large strokes, the brain areas with poor blood flow cannot control pressure well and are more sensitive to high BP. After blood flow is restored, high BP may cause more bleeding and damage, which could explain the worse outcomes seen in these results^[56–59]^.

### Strengths and limitations

Our analysis has several methodological limitations that warrant consideration. First, the clinical heterogeneity across included studies in terms of patient populations (varying stroke types, severity, and treatments), specific SBP targets, and timing of interventions may affect the generalizability of our findings. This heterogeneity is consistent with challenges noted in previous meta-analyses, including Law *et al*^[60]^, who highlighted the difficulty in comparing BP interventions across different clinical contexts. The relatively small sample sizes for some outcomes limit the statistical power and precision of these estimates. This is reflected in the TSA, which indicated that the RISs were not met for several endpoints. Similar limitations were reported by Bath *et al*^[61]^ in their Cochrane review of BP interventions in acute stroke. The variation in BP measurement techniques across studies (attended vs. automated measurements) may introduce measurement bias. Despite these limitations, our analysis has several notable methodological strengths. We conducted a comprehensive systematic review with rigorous methodology, including TSA, to evaluate the robustness and reliability of our findings. The low statistical heterogeneity for most primary outcomes strengthens the consistency of our results across studies. Our rigorous approach to assessing both statistical and reporting biases represents a significant strength of this meta-analysis. The consistent application of Egger’s regression tests across all outcomes, combined with funnel plot examination and TSA, provides a multidimensional assessment of result robustness. Our inclusion of both efficacy and safety outcomes provides a balanced perspective on the risk-benefit profile of intensive BP control, addressing a crucial gap in clinical. Additionally, the exploration of multiple functional outcome measures allows for a more nuanced understanding of how BP management affects different levels of functional recovery.

### Future implications

Several clinical implications can be identified based on our findings. Intensive SBP control consistently improves functional independence (mRS 0–2), indicating that more aggressive BP management may help certain patients achieve independence in fundamental daily tasks. However, the lack of benefit for excellent outcomes (mRS 0–1) combined with an increase in hypotensive events suggests that universal application of intensive targets is not warranted. It seems most prudent to take a personalized approach, taking into account individual patient factors like baseline BP, cerebrovascular status, and comorbidities. This is consistent with the ACC/AHA hypertension guidelines’ recommendations from Whelton *et al*^[62]^, which stress customized BP goals depending on patient characteristics and comorbidities.

### Future recommendations

Our study reveals a number of important knowledge gaps that need more research. First, to ascertain the impact of strict SBP management on mortality, with a focus on cardiovascular versus all-cause mortality, sufficiently powered RCTs are required. Second, patient-level meta-analyses would enable tailored treatment strategies by identifying subgroups that gain the most (or suffer the most) from strict BP management. Moreover, as indicated by conflicting results in different studies, research should concentrate on the best time for BP intervention – that is, whether acute versus chronic care calls for alternative targets. Finally, the integration of home BP monitoring into long-term management strategies deserves further exploration as a method to maintain optimal BP control while minimizing adverse events, following the approach validated by Shimbo *et al*^[63]^ in the SPRINT study.

## Conclusion

This systematic review and meta-analysis concludes that, without raising the risk of death or cerebral bleeding, aggressive SBP management considerably enhances functional independence and early neurological recovery in patients with cerebrovascular illness. Nevertheless, these advantages do not result in superior functional outcomes and come at the expense of more hypotensive episodes. The data backs up a sophisticated, patient-centered strategy for managing BP in cerebrovascular disease that takes into account each patient’s unique characteristics, risk factors, and clinical setting. However, intensive BP lowering increased the risk of hypotensive events in patients, indicating the need for efficient hemodynamic monitoring. These findings highlight that while intensive BP reduction may offer better functional and neurological outcomes, the patients should be monitored, particularly those at risk for impaired cerebral perfusion. Future large-scale, patient-level trials are needed to refine BP targets based on individual risk profiles and imaging characteristics, to guide personalized management strategies after EVT

## Data Availability

All data analyzed during this study are included in this published article and its supplementary materials. Further inquiries can be directed to the corresponding author.

## Research registration unique identifying number (UIN)

Registry: PROSPERO. Registration Id: CRD420251089469. Registration link: https://www.crd.york.ac.uk/PROSPERO/view/CRD420251089469.
